# Advanced seismic attributes applied on resolution-enhanced 2D seismic data for BSR mapping offshore Makran, Pakistan

**DOI:** 10.1038/s41598-026-51110-z

**Published:** 2026-05-05

**Authors:** Muhammad Kamal, Aamir Ali, Tiago M. Alves, Matloob Hussain, Yawar Amin, Muhammad Khan

**Affiliations:** 1 Department of Earth Sciences, Quaid-i-Azam University, Islamabad, 45320 Pakistan; 2 3D Seismic Lab, School of Earth and Environmental Sciences, Cardiff University, Cardiff, CF10 3AT UK; 3 Saudi Aramco, P.O. Box 9060, Dhahran, 31311 Saudi Arabia

**Keywords:** Engineering, Solid Earth sciences

## Abstract

Identifying gas hydrates and associated bottom simulating reflectors (BSRs) in seismic-reflection data requires the use of varied geophysical methods. Re-processed seismic data can primarily highlight seafloor features, but still show inherent signal limitations when resolving small-scale geological features. In contrast, seismic attributes applied to re-processed data have been successfully used in the recognition of hydrocarbon reservoirs. This work tests and applies an integrated seismic-attribute workflow for BSR mapping and qualitative interpretation of BSR-related anomalies, including those reflections commonly interpreted as representing free-gas beneath the BSR in the Makran Accretionary Wedge, offshore Pakistan. Seismic attributes were applied to the resolution-enhanced (re-processed) profile SO122-04a after a structure-oriented data conditioning that used dip-steered median filtering. They were followed by the extraction of amplitude attributes including Pseudo-Relief and Square-Root-of-Energy, and frequency decomposition including Fast Fourier Transform (FFT) and Continuous Wavelet Transform (CWT) evaluated at 20, 40, and 60 Hz. The results in this work show that Pseudo-Relief emphasizes the geometric expression of seismic anomalies and BSRs by illuminating their curvature and shape, while Square-Root-of-Energy highlights spatial variations in reflection energy across BSR intervals. FFTs and CWTs show comparatively stronger amplitude expression of BSRs and underlying reflections in the 40 and 60 Hz frequency bands. Finally, RGB color blending aids the visual identification of BSR-related anomaly zones (indicators of potential hydrate/free-gas systems), further supporting attribute-based mapping for the interpretation of BSR-related anomalies. Interpretations are qualitative in the absence of independent calibration data.

## Introduction

Gas hydrates comprise natural gas in a crystalline solid state that forms naturally in deep-water areas of continental margins and in permafrost regions, i.e. where low-temperature and high-pressure conditions are encountered^1^. In conventional seismic reflection sections, gas hydrates are not imaged directly; rather their occurrence is commonly inferred from indicators such as Bottom Simulating Reflectors (BSRs) at a relatively shallow depth below the seafloor, as they respond to the acoustic contrast between gas hydrates and free-gas below^2,3,4,5,6^. In practice, BSRs form a distinct seismic reflection that is correlated with the base of gas hydrate stability in sediment^7,8,9,10^. BSRs are usually associated with: (a) diagenetic boundaries (fronts), representing intervals where diagenetic phenomena occurred or occur at present, and where new cement is generated; and (b) impedance contrasts across the base of the hydrate stability zone, where hydrate-bearing sediments overlie gas-charged sediments. Importantly, seafloor-parallel reflections can also arise from non-hydrate causes (e.g., diagenetic fronts), so BSR-like events are non-unique.

The identification of BSRs is based on a detailed interpretation of seismic data, namely on the recognition of local polarity reversals in near-seafloor sediment with respect to the seafloor reflection. The reverse-polarity reflectors that compose BSRs mirror the seafloor topography and often crosscut any dipping sediment, giving rise to their name^11,12,13^. Gas hydrate systems and their associated BSRs are typically investigated using high-quality seismic data; in our case we use a resolution-enhanced (re-processed) 2D line that images porous sediment near the modern seafloor. However, because the presence of hydrates is difficult to confirm from seismic-reflection profiles alone, additional geophysical methods are commonly used where available. Seismic attribute analysis is a particularly useful tool for delineating BSR-related anomalies and reflector geometry, thereby assisting in qualitative interpretation, but it does not provide quantitative hydrate or free-gas estimates without independent calibration^14,15^. We nevertheless stress the fact that accurately understanding BSR occurrence below the seafloor can be relevant for geohazard considerations; hydrate dissociation driven by climatic, oceanographic, or tectonic changes may contribute to overpressure development near the seafloor^16^. These locally overpressured strata are prone to become unstable, potentially triggering catastrophic submarine landslides and associated tsunamis^17,18,19,20,21^.

BSR-like, seafloor-parallel reflectors can be misidentified because similar events may arise from diagenetic fronts, tuning/interference, multiples, stratigraphic interfaces, or processing/migration footprints. Attribute responses can also be influenced by local wavelet variability, residual noise, and bandwidth limitations. To reduce ambiguity, we interpret BSRs using multiple criteria (cross-cutting relationships, reverse polarity relative to the seafloor, and spatially coherent attribute expressions) and use attributes as comparative visualization tools, rather than standalone evidence. Because no wells/cores or velocity-inversion constraints are available along SO122-04a, interpretations in this study remain qualitative.

Post-stack and pre-stack attributes are techniques used over the last few decades to map subsurface gas hydrates^22^. Such attribute-based characterization has been applied in hydrate/free-gas studies since early work^23^. Curvature, dip-azimuth, seismic coherency, similarity, and physical attributes such as instantaneous amplitude, instantaneous phase, and instantaneous frequency, have been effective in imaging geological structures and qualitatively highlighting reflection-character variations^24^. In fact, the presence of sub-surface gas hydrates is often associated with a diminished amplitude, heightened frequency, and reduced sweetness of seismic reflections. In contrast, the presence of a free-gas zone (FGZ) below BSRs has been reported in many hydrate provinces and is commonly associated with boosted seismic amplitudes, lower apparent frequencies, and increased sweetness^24^. Accordingly, authors such as ^14^ have previously discussed the relationship between amplitude and gas hydrates occurrence and associated indicator strength. Spectral decomposition techniques were used by^25^ to distinguish between gas hydrates and FGZs. BSRs were identified by ^26^ using L_1_ norm deconvolution and post-stack seismic properties such as instantaneous frequency, instantaneous envelope, amplitude envelope, first derivative, and instantaneous phase. By analyzing seismic attributes such as attenuation, frequency, reflection strength, and using techniques such as principal component analysis (PCA) and self-organizing maps (SOM), one can identify the characteristic amplitude and frequency patterns associated with the presence of gas hydrates in sub-surface strata^27^.

Frequency and spectral decomposition attributes are widely used for the characterization of hydrate/gas-free systems by analyzing the frequency-dependent reflection behavior and helping delineate BSR-related anomalies. These attributes are extracted from seismic data and offer insights into the relative contribution of different frequency components in a given seismic dataset^28,29^. More importantly, frequency and spectral decomposition attributes can help reveal BSR-related reflectors and associated amplitude–frequency anomalies, particularly where conventional displays are limited by bandwidth and signal-to-noise (S/N) ratio. Such attributes have been used in the energy industry for decades to locate strata that are highly porous or permeable and/or record lateral structural changes (dip direction, strike), variable continuity, local stratigraphic pinch-outs, and to collect a variety of other stratigraphic information^30^.

While these approaches are well established, previous applications often examine individual attributes in isolation or rely on conventionally processed data and external calibration to support interpretation. The key contribution of this work is the integrated and reproducible application of structure-oriented conditioning (dip-steered median filtering), amplitude attributes (Pseudo-Relief; Square-Root-of-Energy), and frequency decomposition (FFT and CWT evaluated at 20, 40, and 60 Hz) to a resolution-enhanced 2D profile (SO122-04a) previously re-processed in^31^. Our contribution is therefore a qualitative assessment of attribute performance and internal consistency for BSR mapping in a data-sparse setting.

**Fig. 1:**
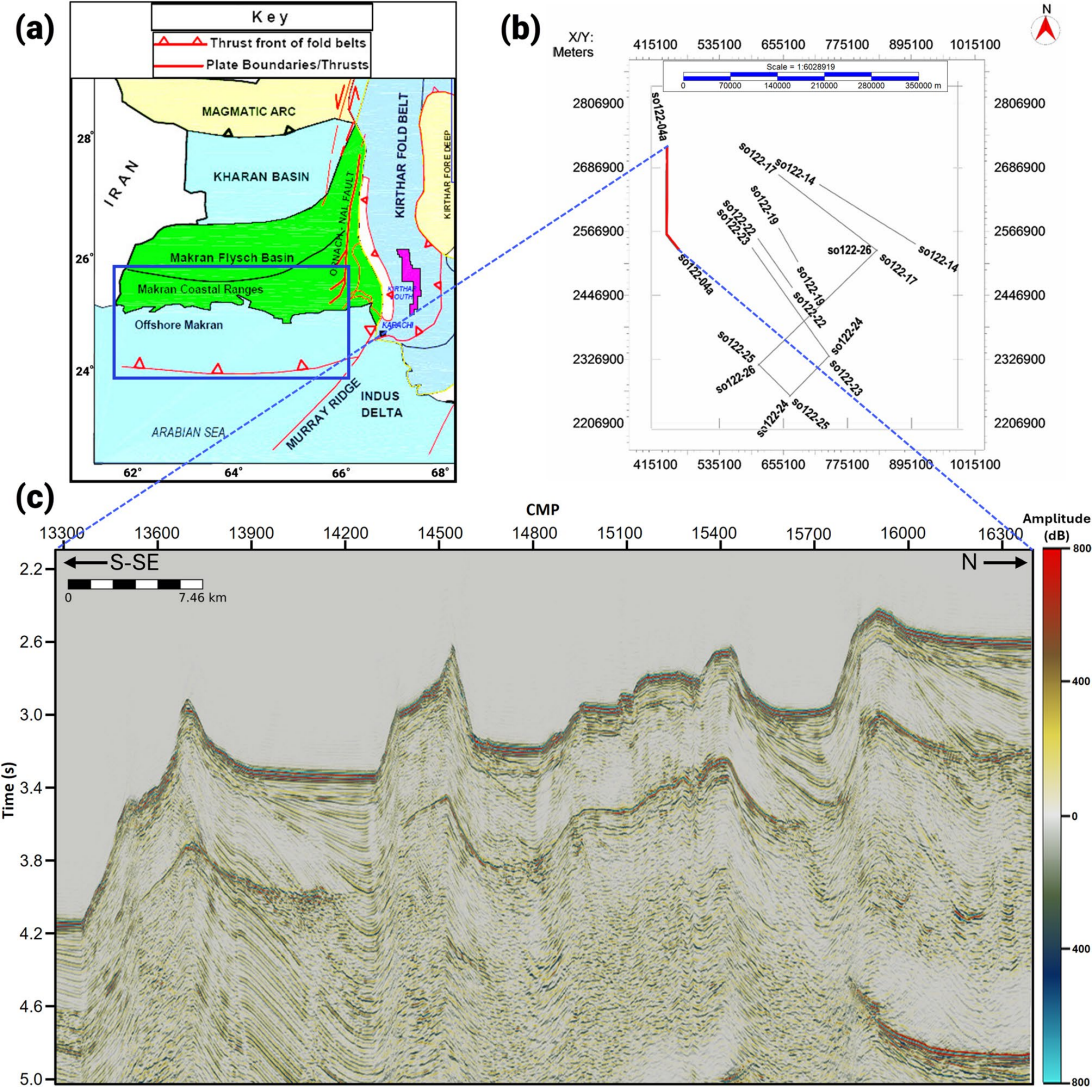
(**a**) Location of the study area offshore Makran, southern Pakistan, as highlighted by the blue rectangle (modified after^31^. (**b**) Basemap showing the location of seismic profiles acquired offshore Makran. (**c**) Seismic profile SO122-04a selected for the in-depth attribute analysis undertaken in this work. Its location is highlighted in red in Fig. 1b. Seismic profile SO122-04a is modified from Ref. ^13^. Figures were generated using Kingdom Suite^x^ (Version 2017, IHS Markit- https://ihsmarkit.com).

This study develops a comprehensive seismic-attribute analysis of seismic profile SO122-04a acquired offshore Makran (Fig. 1a), after it was re-processed using the method proposed in ^31^. This seismic profile was preferred to others because it images a pronounced BSR that is associated with the presence of near-seafloor gas hydrates, as reported and interpreted in previous studies^4,31^. The imaged BSR fulfills the typical acoustic characteristics of gas hydrates in near-seafloor strata, i.e. it typically crosscuts sedimentary strata, shows inverse polarity relative to the seafloor, and broadly follows seafloor bathymetry.

Given the lack of independent calibration data at SO122-04a, we focus on qualitative delineation of BSR-related anomalies and evaluation of attribute performance rather than quantitative hydrate assessment. Our approach aims to leverage commonly used seismic attributes by enhancing the resolution of subsurface features in seismic profile SO122-04a. The study evaluates a combined set of established attributes to support the qualitative interpretation of BSR-related anomalies on SO122-04a. In summary, this work addresses the following research questions:

1. Can amplitude-based attributes, applied to a processed seismic profile conditioned with dip steered median filtering, assist in the delineation of the BSR?
2. Can seismic-frequency decomposition using the Fast Fourier Transform (FFT) and Continuous Wavelet Transform (CWT) reveal frequency-dependent variations in seismic features associated with BSRs and underlying reflections that may indicate the presence of free-gas beneath the BSR?
3. Can the application of average- and median-amplitude attributes reveal seismic anomalies associated with gas hydrate indicators?

## Geological setting

### Geological setting of offshore Makran

The offshore part of the Makran coincides with the northern boundary of the Gulf of Oman’s abyssal plain and is characterized by its relatively narrow continental shelf and slope^32,33^. Under Makran’s offshore region, the Arabian Plate is subducted at a rate of ~ 4 cm per year beneath the Eurasian Plate, forming the large E-W-striking Makran Subduction Zone, a major bathymetric and tectonic feature that spans the shores of southern Pakistan and Iran^34^ (Fig. 1a). Near the Makran Subduction Zone occurs one of the world’s largest accretionary prism, with a sediment thickness of up to 7.5 km^33^. Regional seismic profiles show an ocean-continent boundary topped with c. 5 km of sediment^35,36^, and these sediments are mostly horizontal on the Makran abyssal plain. However, they are abruptly deformed into a 50 km-wide fold-and-thrust belt as they approach Pakistan’s coast. Importantly, only the top 2.5 km of the sedimentary sequence are folded as they are detached at the base by a regional décollement. As revealed by offshore drilling, the majority of this folded sequence consists of Early Miocene and younger sediments^32^.

### Nearshore stratigraphy

Various structural and depositional features occur along the Makran margin and reflect active deformation, erosion, and sedimentation^37^. Of particular relevance to gas hydrate systems, the nearshore and offshore parts of the Makran are characterized by important mud volcanism, which indicates focused fluid expulsion associated with deformation and dewatering of the accretionary prism^37^. These pathways can influence the distribution of shallow gas and the occurrence of BSR-related anomalies observed in seismic data.

## Data and methods

### Seismic dataset and regional context

The multichannel seismic data used in this work were acquired during a campaign carried out by the Federal Institute for Geosciences and Natural Resources (BGR, Hannover). This campaign was organized in collaboration with GEOMAR (Kiel), the National Institute of Oceanography (NIO, Karachi), and the Hydrocarbon Development Institute of Pakistan (HDIP)^38^. It focused on the investigation of the Makran offshore area in terms of conventional hydrocarbon resources, as well as understanding crustal structure and the local occurrence of gas hydrates as important near-seafloor geohazards^38^. The campaign gathered evidence for hydrate deposits and free methane gas, thus justifying the important offshore gas seepage that is recorded along the continental shelf and slope offshore Makran^39^.

### Prior processing and resolution enhancement of SO122-04a

The work from^38^ documented that the acquired seismic reflection data were processed using the available standard methods but, as a result, they also reveal a poor S/N ratio. In a first instance, this resulted in a relative lack of confidence when mapping laterally extensive Bottom-Simulating Reflectors (BSRs). Consequently,^24^ proposed and executed an advanced, amplitude-preserving processing workflow on the same seismic dataset and significantly improved its vertical and horizontal resolutions. The method in^24^ resulted in an enhancement of vertical resolution through the inversion of direct arrivals, and significant improvements in horizontal resolution. Both improvements required the selection of an optimal migration algorithm and velocity model^24^.

### Profile selection and overview of attribute workflow

**Fig. 2:**
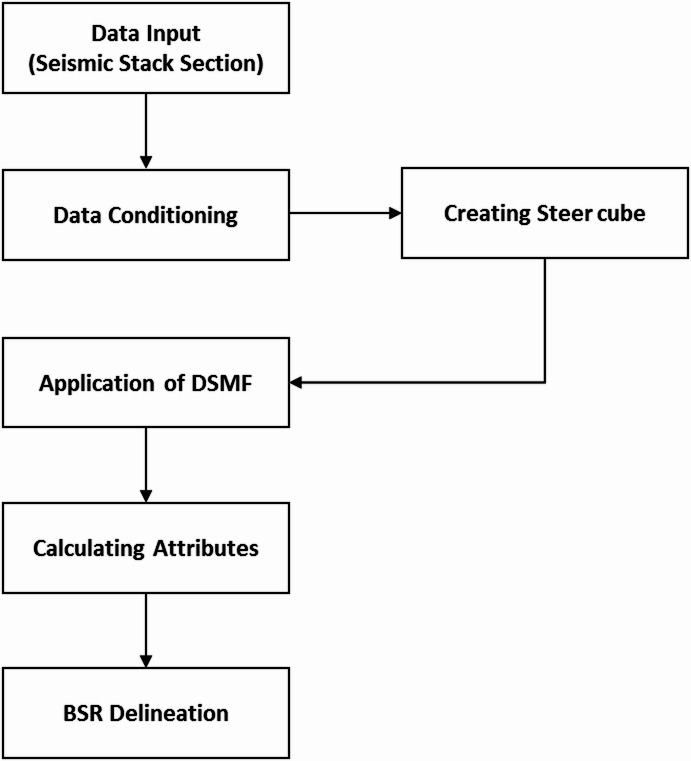
Diagram illustrating the different stages involved in attribute analysis. This analysis included data preparation, data conditioning, attribute extraction, attribute visualization and interpretation, which led to a precise delineation of a BSR in the study area.

Seismic profile SO122-04a, re-processed using the methodology proposed in^24^, was selected for this work as it presents a continuous, reflective BSR (Fig. 1). The locations of all available seismic profiles from offshore Makran are shown in Fig. 1b, whereas the originally processed seismic profile SO122-04a is shown in Fig. 1c. The workflow summarizing the stages of attribute analysis adopted in this study is shown in Fig. 2.

### Frequency-band selection for FFT/CWT decomposition

Frequency bands (20, 40, and 60 Hz) were selected based on the bandwidth characteristics reported in ^31^ and were used consistently for FFT/CWT comparisons. The amplitude spectrum of the re-processed data shows dominant energy broadly between 10 and 50 Hz (with a peak around 20–35 Hz), while amplitudes decrease markedly beyond 50–60 Hz. We therefore selected 20 Hz (low), 40 Hz (dominant/intermediate) and 60 Hz (upper usable band) to compare frequency-dependent expression of the BSR interval using both FFT and CWT.

### Attribute-analysis workflow overview

Seismic data conditioning refers to the processing steps used to suppress noise and stabilize reflection character prior to interpretation and attribute analysis. Because seismic attributes are particularly sensitive to incoherent (random) noise and discontinuous reflector geometry, structure-oriented pre-conditioning is commonly applied to improve reflector continuity and reduce seismic artefacts^40,41^. Accordingly, the attribute workflow in Fig. 2 was implemented in sequential steps: (i) structure-oriented pre-conditioning using a dip-steered median filter ("Pre-conditioning / dip-steered median filtering" section), (ii) amplitude-attribute computation ("Amplitude-attribute computation" section), (iii) frequency decomposition using FFT and CWT ("Frequency-based attribute computation" section), (iv) RGB blending of decomposed components ("RGB color blending of decomposed components" section), and (v) multi-frequency integration using average and median responses ("Integration of frequency attributes" section). These steps are described below in the order they were applied.

### Pre-conditioning / dip-steered median filtering

**Fig. 3:**
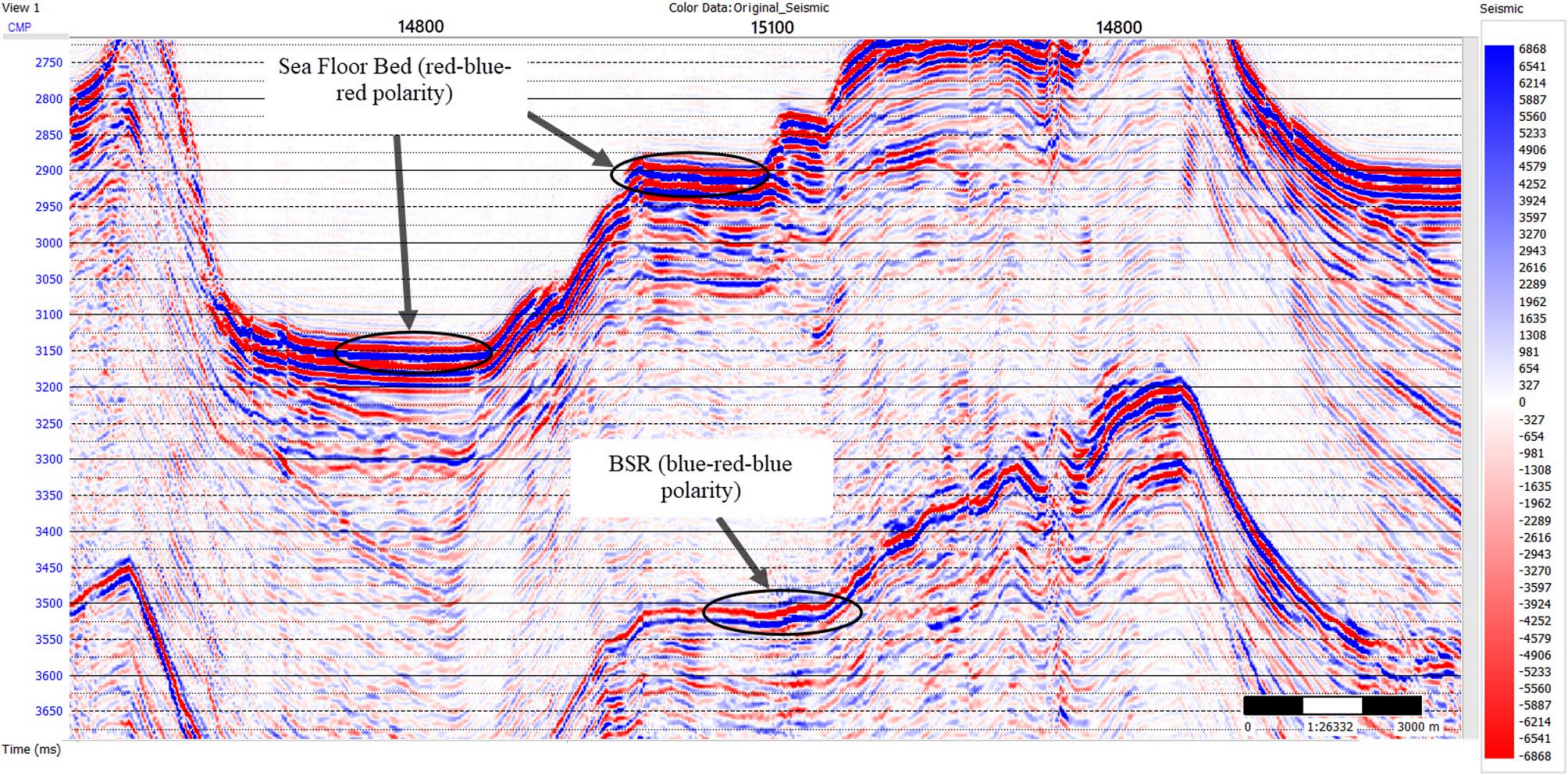
Polarity QC on profile SO122-04a (wiggle/variable-area display). Representative seafloor reflections show a red–blue–red character, while the interpreted BSR exhibits opposite polarity (blue–red–blue) at the highlighted locations, consistent with the reverse-polarity BSR criterion used in this study.

Pre-conditioning filters such as dip-steered median filter (DSMF), structure-oriented filtering, edge-preserving smoothing, and structural smoothing can improve reflector continuity and reduce incoherent noise in seismic data. Seismic attributes can be sensitive to lateral changes in waveform shape and amplitude between traces, and their response can be affected by local dip across discontinuities^40,41^. The seismic section analyzed here is the final post-stack profile reported in^24^ in its re-processed version, and we do not reprocess field data in the present study. Therefore, attribute responses are evaluated on this published stack and are interpreted qualitatively. Processing steps and any amplitude scaling applied during stacking/migration are those documented in ^24^ - their legacy workflow includes AGC with a 750 ms window, whereas the reprocessing workflow focuses on de-signature, denoising, and migration/velocity refinement. Because the reverse-polarity BSR criterion is central to our interpretation, we include a wiggle/variable-area inset (SEG normal polarity) illustrating the seafloor and BSR wavelet character and confirming opposite polarity between the two reflectors (Fig. 3). Polarity comparison is based on wiggle/variable-area traces (not color fill), using SEG normal convention.

The DSMF is a spatial filter that preserves the edges and discontinuities of seismic features, eliminating random noise and increasing the lateral continuity of reflections. It results in the smoothing of the edge effect and an increase in S/N ratio^42,43^. Dip-steer algorithms estimate the local dip direction of seismic events^40,44^. Dip steering can be described as the process of following a dip from one point on a seismic trace to adjacent traces^40^. In practice, this trace-to-trace approach functions similarly to a local auto-tracker, helping to identify laterally coherent events across neighboring traces^45^.

**Fig. 4:**
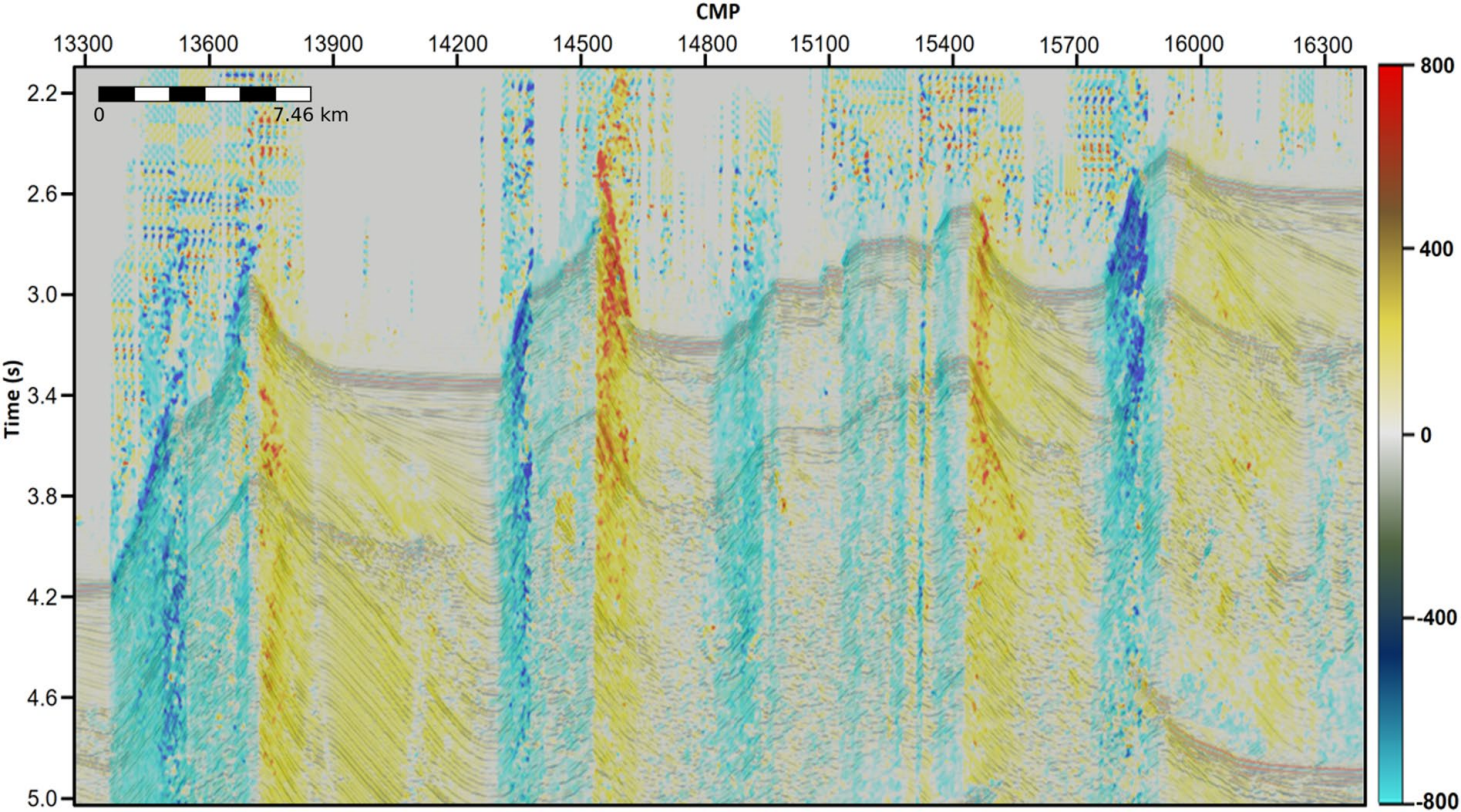
Dip-steer computed for seismic profile SO122-04a and used to steer the median filter. The blue and red indicate opposite dip directions along the profile.

Although SO122-04a is a 2D profile, dip estimation and steering were computed along the profile using a structure-oriented (PCA-based) dip field to guide the median filter. The resulting dip field used for steering is shown in Fig. 4, where the color contrast indicates opposite dip directions along the profile. In this context, dip steering refers to the trace-to-trace tracking of locally coherent reflection events along the profile, using the estimated local dip to orient the filtering window parallel to reflectors. This structure-oriented approach suppresses incoherent noise while preserving reflector terminations and small discontinuities that are important for BSR interpretation. We used PCA-based dip estimation because it yields smoother and more stable dip estimates under variable signal-to-noise conditions, reducing sensitivity to noise compared with simple gradient-based dip estimates^40,44^.

**Fig. 5:**
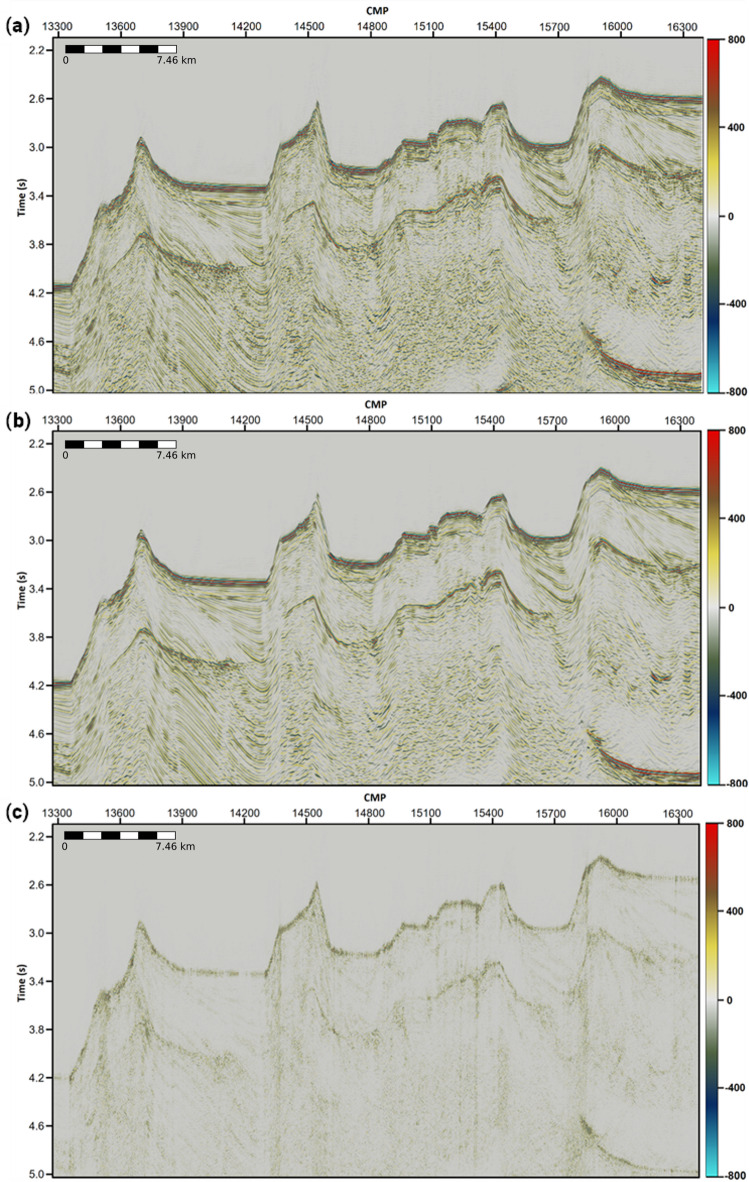
Comparison of seismic profile SO122-04a before and after structure-oriented DSMF. (**a**) Original stacked seismic section. (**b**) Conditioned section after applying a PCA-based dip field to steer the median filter (parameter set [3, 3]). (**c**) Difference image (original minus conditioned), highlighting energy attenuated by the structure-oriented filter. The DSMF suppresses incoherent energy and improves reflector continuity while preserving major reflector geometries relevant for BSR interpretation.

**Fig. 6:**
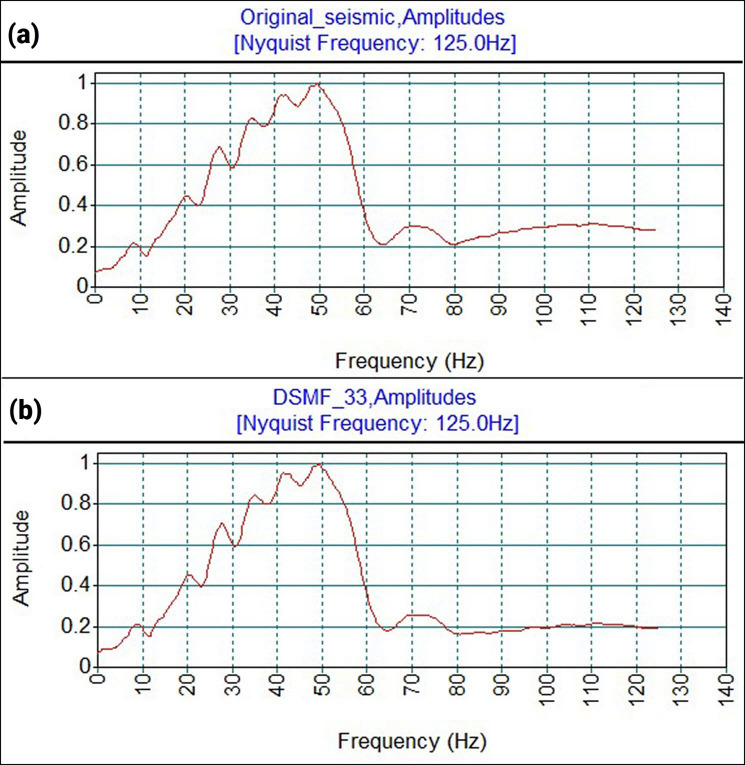
Amplitude spectrum comparison for seismic profile SO122-04a computed over the same 2.2–5.0 s time window. (**a**) Original stacked data. (**b**) Conditioned data after PCA-guided dip-steered median filtering (DSMF parameter set [3,3]). The main signal bandwidth is broadly preserved after conditioning, while the post-conditioning spectrum shows a reduced high-frequency tail, consistent with suppression of incoherent noise rather than artificial spectral boosting (Nyquist frequency = 125 Hz).

The primary reason for selecting a PCA-based dip estimation is that it provides a smooth, stable estimate of local reflector dip, which is required to steer the structure-oriented (dip-steered) median filter along the profile. This dip parameter is crucial for understanding the orientation and structural characteristics of subsurface features along the profile. Additionally, PCA is known for its computational efficiency, allowing for a swift data processing, and is also effective in the application of structure-oriented smoothing to raw seismic data. To achieve this conditioning, we used three different sets of filtering parameters [2, 2], [3, 3], and [5, 5], where the two values refer to the lateral (trace) and vertical (time) neighborhood used to steer the filter. In each case, the PCA algorithm was applied to the seismic data to extract smoother dip estimates for seismic reflections. After experimentation, the filtering parameter set [3, 3] was chosen as the optimal among all the options tested (Fig. 5). To assess whether this conditioning altered the data’s frequency content, we compared amplitude spectra from the pre- and post-conditioned profiles over the same analysis window (2.2–5.0 s TWT below seabed) using identical spectral parameters (Fig. 6).

In addition to this spectral check, we quantified the impact of seismic conditioning. We computed a robust noise-suppression metric based on the incoherent residual energy. For each trace, the amplitude is denoted by $x \left(t\right)$, and a laterally coherent predictor was estimated using a rolling-median filter across adjacent traces (i.e. excluding the central trace) according to^46,47,48^. Half-window will be $w= 3,$ i.e., a 7-trace window, as shown in Eq. 1:

1 $$\widehat{x}_{i}\left(t\right)=median\left\{x_{i}\left(t\right):\:j\:\in\:\:\left[i-w,\:i+w\right],\:j\:\ne\:\:i\:\right\}$$

The incoherent (residual) component is subsequently estimated by Eq. 2:

2 $$r_{i}\left(t\right)=x_{i}\left(t\right)-\widehat{x}_{i}\left(t\right)$$

For a trace-specific analysis gate [$t_{0,i}, t_{1,i}$], the RMS residual energy is computed by Eq. 3 below^48^:

3 $$\:\mathrm{RM}{\mathrm{S}}_{\mathrm{r}}\left(i\right)=\sqrt{\frac{1}{{N}_{i}}{\sum\:}_{t\in\:\left[{t}_{0,i},\hspace{0.17em}{t}_{1,i}\right]}{r}_{i}{\left(t\right)}^{2}}$$

We summarize conditioning performance as the median reduction in residual RMS (Eq. 4):

4 $$\varDelta\:\%=100\times\:\left(1\:-\frac{median\left(RMS_{r},post\left(i\right)\right)}{median\left(RMS_{r},pre\left(i\right)\right)}\right)$$

The residual $r_{i}\left(t\right)$ represents incoherent, noise-like energy not explained by laterally persistent signal. Because data above the seabed are undefined (blanked), a traced seabed horizon was used to define trace-specific valid gates below the seabed. Residual RMS was computed within (i) a global sub-bottom window from 2.2 to 5.0 s TWT below the seabed and (ii) a BSR-focused window from 3.3 to 3.9 s TWT below the seabed. This comparison used the common set of traces available in both datasets (index-matched to the conditioned profile length, *n* = 1626 traces). Summary statistics (mean, standard deviation, median, and interquartile range) were computed across valid traces, and median residual-RMS reduction was used as the main QC indicator (Table 1).

**Table 1:** QC metrics for seismic conditioning using incoherent residual RMS energy. Residuals use a rolling-median predictor (± 3 traces) and trace-specific gates below the seabed mute. Results are shown for global (2.2–5.0 s) and BSR (3.3–3.9 s) windows.

GLOBAL QC (2.2–5.0 s TWT below the seabed)					
Gate definition	t0 = max (seabed, 2.2 s), t1 = 5.0 s				
Valid traces / total (fraction)	1572 / 1626 (96.68%)				
Median residual-RMS reduction	29.16%				
Metric	Mean	Std	Median	IQR	N
RMS_incoh_pre	2,156.24	1,083.32	1,966.44	1,766.28	1572
RMS_incoh_post	1,639.72	1,057.68	1,393.08	1,621.46	1572
ΔRMS_incoh (post–pre)	-516.52	565.30	-444.12	460.16	1572
BSR QC (3.3–3.9 s TWT below seabed)					
Gate definition	t0 = max (seabed, 3.3 s), t1 = 3.9 s				
Valid traces / total (fraction)	1481 / 1626 (91.08%)				
Median residual-RMS reduction	36.40%				
Metric	Mean	Std	Median	IQR	N
RMS_incoh_pre	1,895.90	1,262.24	1,527.09	1,362.80	1481
RMS_incoh_post	1,369.07	1,163.72	971.17	1,198.96	1481
ΔRMS_incoh (post–pre)	− 526.83	622.49	-426.11	529.49	1481

### Amplitude-attribute computation

Seismic attribute analyses can help identify BSRs in seismic data. This involves extracting quantitative measures from the seismic trace and interpreting them to gain insights into the subsurface geology and potential geological resources. Seismic attributes have been demonstrated as effective in highlighting subsurface structures and are commonly employed to identify probable hydrocarbon reserves^25^. A focused use of these attributes can help reveal lithological and structural contrast in buried strata. Information regarding spatial variability in seismic data can provide valuable insights into lithological and stratigraphic heterogeneity in reservoirs^50^. Accordingly, a set of amplitude-based attributes were used to increase the chance of gas hydrate detection, adding value to the interpretation of BSRs and underlying reflections commonly interpreted as possible free-gas indicators.

Amplitude-based attributes are often used in the acoustic characterization of gas hydrates^24,50^. The presence of gas hydrates can have a significant effect on seismic response because of the contrast in acoustic impedance between hydrate-carrying intervals and the surrounding sediment. Amplitude is the most often used parameter in reflection seismology for lithological interpretation and reservoir prediction. In addition, the amplitude of seismic reflections can be quantified using amplitude-based attributes, which can highlight the presence of gas hydrates. Geological elements that influence amplitude include lithological changes, different fluid phases, the nature of the host sediment, the presence of unconformities, strata tuning effects, and stratigraphic heterogeneity^50^.

**Fig. 7:**
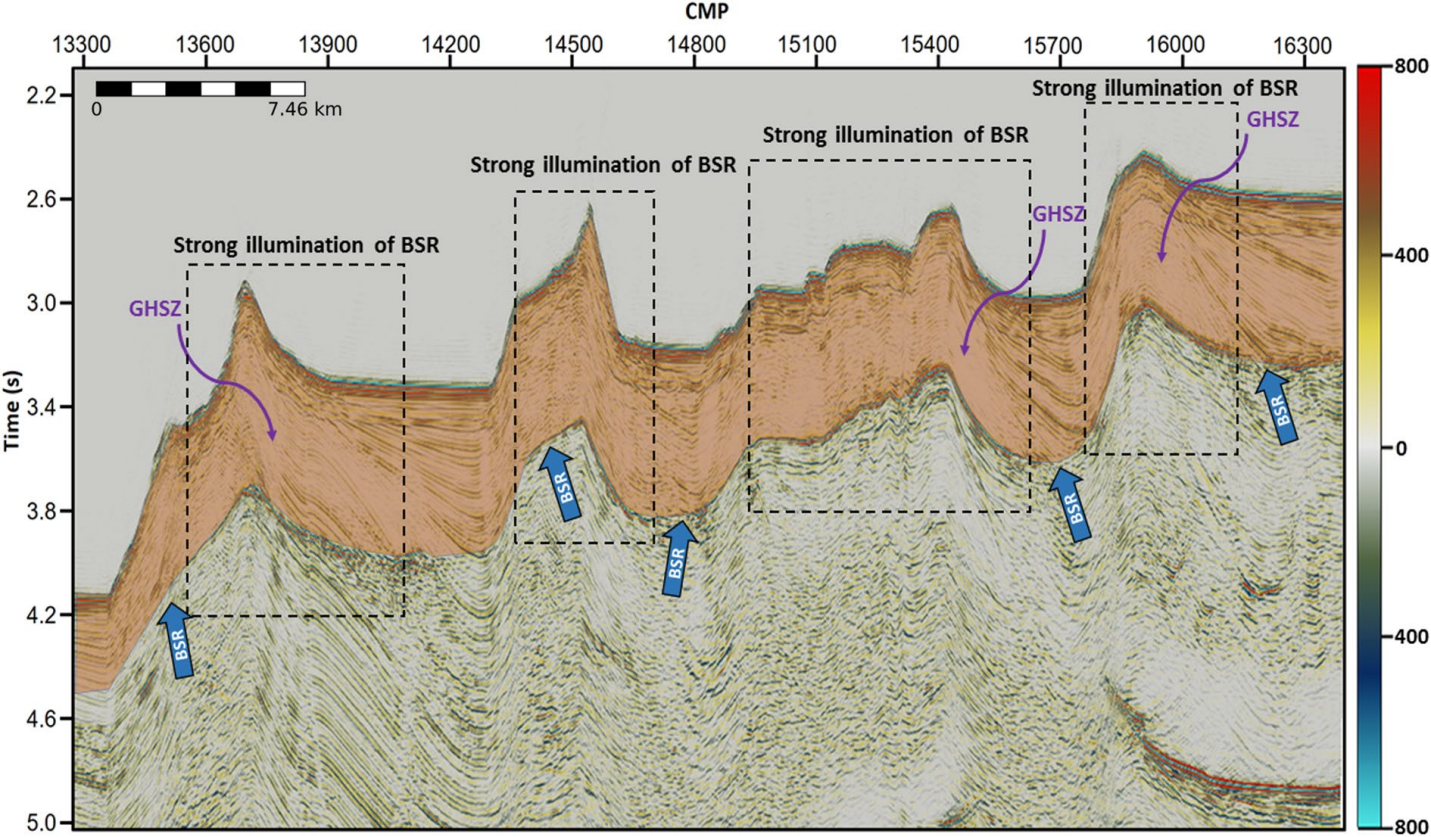
Interpretation of the dip-steered median-filter conditioned seismic profile SO122-04a, with the BSR and the GHSZ highlighted. Zones where the BSR is expressed more clearly after conditioning are highlighted, indicating intervals where hydrate indicators are interpreted along the seismic profile.

Gas hydrate reservoirs can often produce distinctive seismic anomalies, which are manifested as localized regions of anomalously high or low amplitudes compared to the surrounding sediment. Amplitude anomaly attributes highlight these deviations and can be consistent with spatial variations in gas hydrate occurrence and associated free-gas effects^22,24,51^. Knowing this, the Pseudo-Relief attribute is a seismic attribute that helps to highlight BSRs by emphasizing their curvature and shape. This attribute is obtained by the application of a Hilbert transform, i.e. a − 90° phase rotation, to the Energy attribute when computed within a short time window^52^. It generates outcrop-like images and facilitates the detection of both faults and horizons^53^. Compared to the standard amplitude image, it highlights the continuity or discontinuity of reflectors, thereby enhancing steep discontinuities and fault zones in the seismic data^53^. Accordingly, we computed Pseudo-Relief on the conditioned SO122-04a profile to help reveal reflector continuity and highlight curvature-based expression of the BSR interval (Fig. 7).

**Fig. 8:**
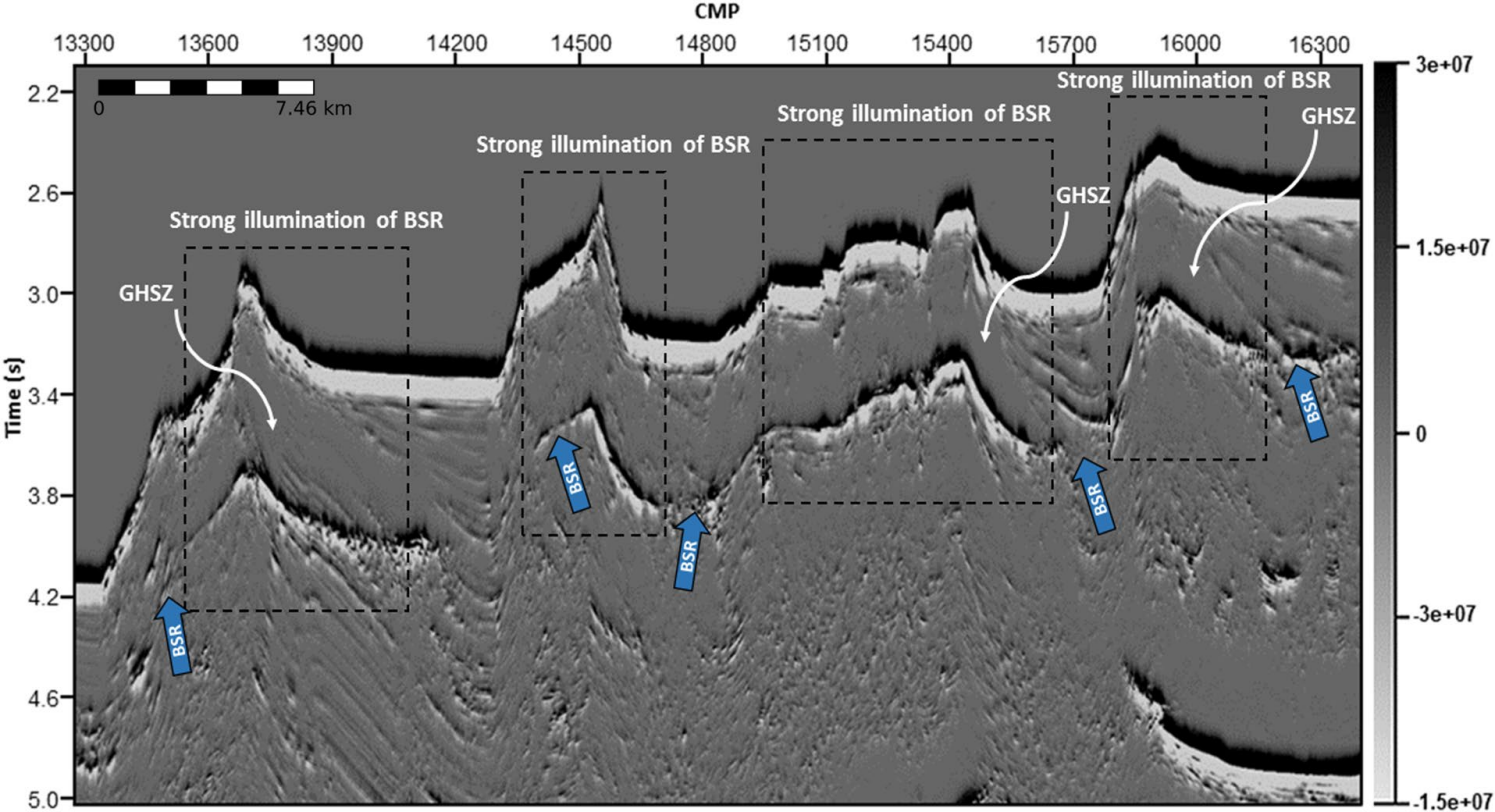
Pseudo-Relief attribute applied to seismic profile SO122-04a highlighting the geometric expression of the BSR by emphasizing reflector curvature and shape. Areas of stronger BSR expression occur near the tops of structural highs.

Square-Root-of-Energy is used in seismic data interpretation to highlight subsurface features such as faults, fractures, and stratigraphic discontinuities . This attribute estimates the root-mean-square of seismic amplitudes over a time window^54^. The seismic amplitudes are squared and subsequently averaged over the selected time window, and the square root of this averaging is then calculated. The process ensures that all amplitudes are positive, eliminating polarity differences in the seismic signal and emphasizing higher-frequency components of the seismic data . The attribute is particularly useful for revealing contrasting bedding geometries and sharp lateral variations, making it helpful in the identification of structural features and stratigraphic boundaries . We also computed Square-Root-of-Energy on the same conditioned profile to visualize the reflection-energy variations across the BSR interval (Fig. 8).

### Frequency-based attribute computation

Frequency attributes are routinely used in gas hydrate detection by isolating and categorizing seismic events based on their frequency^15,28,29^. In practice, frequency attributes separate the seismic signal into its fundamental frequencies through spectral decomposition allowing an analysis of the phase and amplitude at different wavelengths. Importantly, the phase component only identifies lateral discontinuities, whereas the amplitude component is more sensitive to lateral discontinuity and to variations in strata thickness^51^. To create spectrally decomposed sections, recorded seismic traces can be transformed from their time or depth domain to the frequency domain using a variety of methods.

This study made use of the FFT and the CWT spectral decomposition to extract the frequency components of seismic profile SO122-04a at potential gas hydrate locations. The FFT is a computationally efficient method for generating a Fourier transform and is used for investigating numerous signal characteristics in the frequency domain. In parallel, the CWT method is used to detect and explore hidden complex reservoir structures. One of the advantages of using these two techniques is their ability to achieve optimum temporal resolution for higher and lower frequencies^56,57^. The FFT and CWT decompositions were computed on the conditioned SO122-04a profile to ensure consistent comparison with the amplitude attributes.

Spectral decomposition was performed on the conditioned amplitude section using both a sliding-window FFT (STFT-style) and a CWT. For the FFT-based decomposition, we used a 56 ms sliding time gate (± 28 ms about the central sample); within each gate the FFT was computed, and the window advanced sample-by-sample along each trace to generate time-varying frequency slices. Output frequency components were extracted at 20 Hz, 40 Hz, and 60 Hz (frequency sampling step = 1 Hz). For the CWT decomposition, we used a Gaussian mother wavelet and sampled the same target frequencies. The Gaussian wavelet was chosen because it provides comparatively strong time localization in the time–frequency representation, which can yield sharper spectral slices and reduce visual time-smearing of thin or closely spaced reflectors relative to longer fixed-window Fourier transforms; we treat this as a visualization advantage rather than a unique physical indicator.

**Fig. 9:**
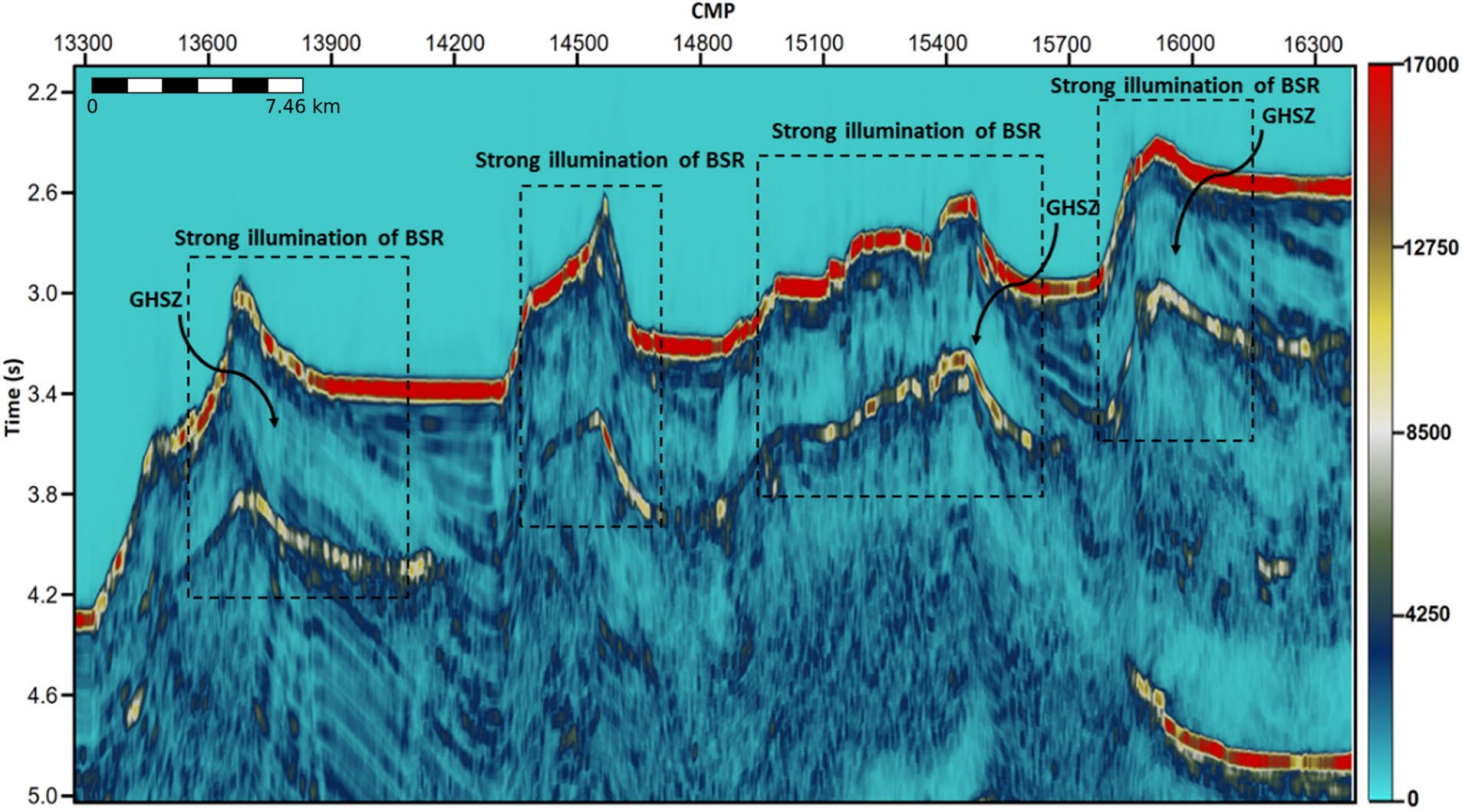
Square-Root of Energy attribute applied to seismic profile SO122-04a highlighting energy variations associated with the BSR interval. The attribute displays the BSR as high-energy features, depicted by yellow and red colors.

**Fig. 10:**
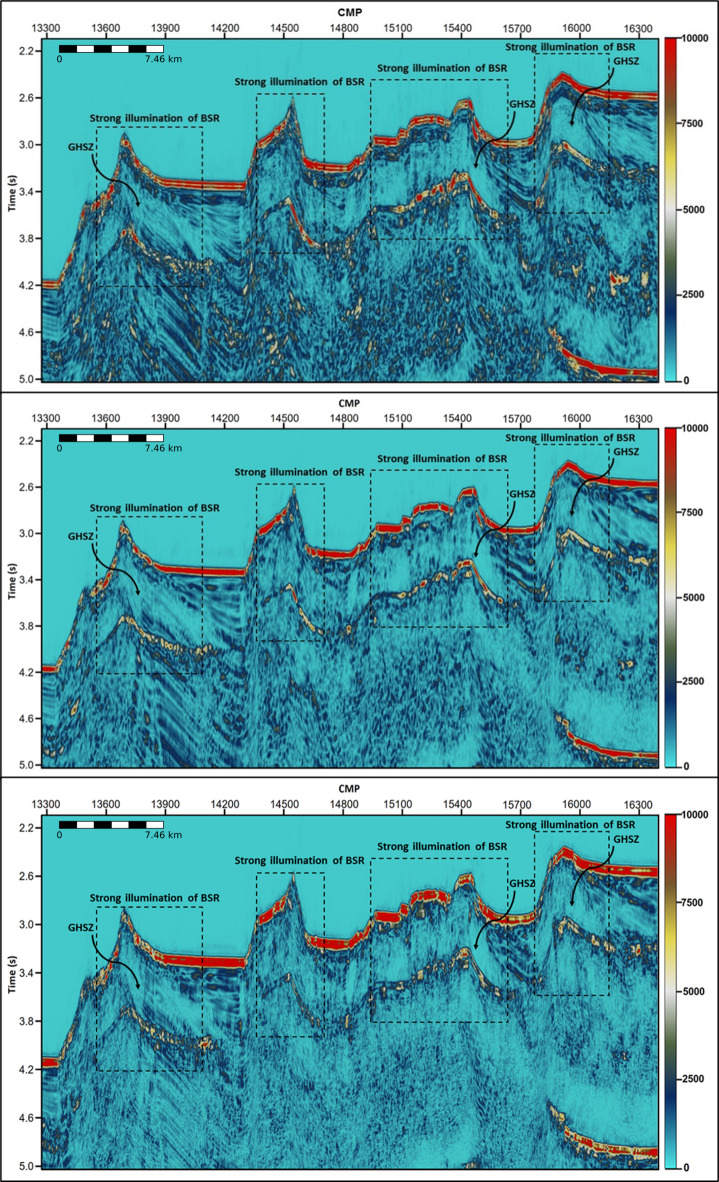
FFT-based spectral decomposition of seismic profile SO122-04a at (**a**) 20 Hz, (**b**) 40 Hz, and (**c**) 60 Hz. The 20 Hz component emphasizes the lateral continuity of the BSR expression, whereas the 40 and 60 Hz components display more distinct reflector patterns within the BSR interval and adjacent reflectors, enabling comparison of frequency-dependent BSR-related anomaly patterns across the usable bandwidth.

We extracted frequency slices at 20, 40, and 60 Hz (Frequency-band selection for FFT/CWT decomposition" section) to examine frequency-dependent variations in BSR expression and associated underlying reflections. We first applied FFT-based spectral decomposition to extract attribute responses at 20, 40, and 60 Hz (Fig. 9). We then applied CWT-based spectral decomposition using the same frequency bands to enable a direct comparison of time–frequency behavior across the BSR interval (Fig. 10).

### RGB color blending of decomposed components

**Fig. 11:**
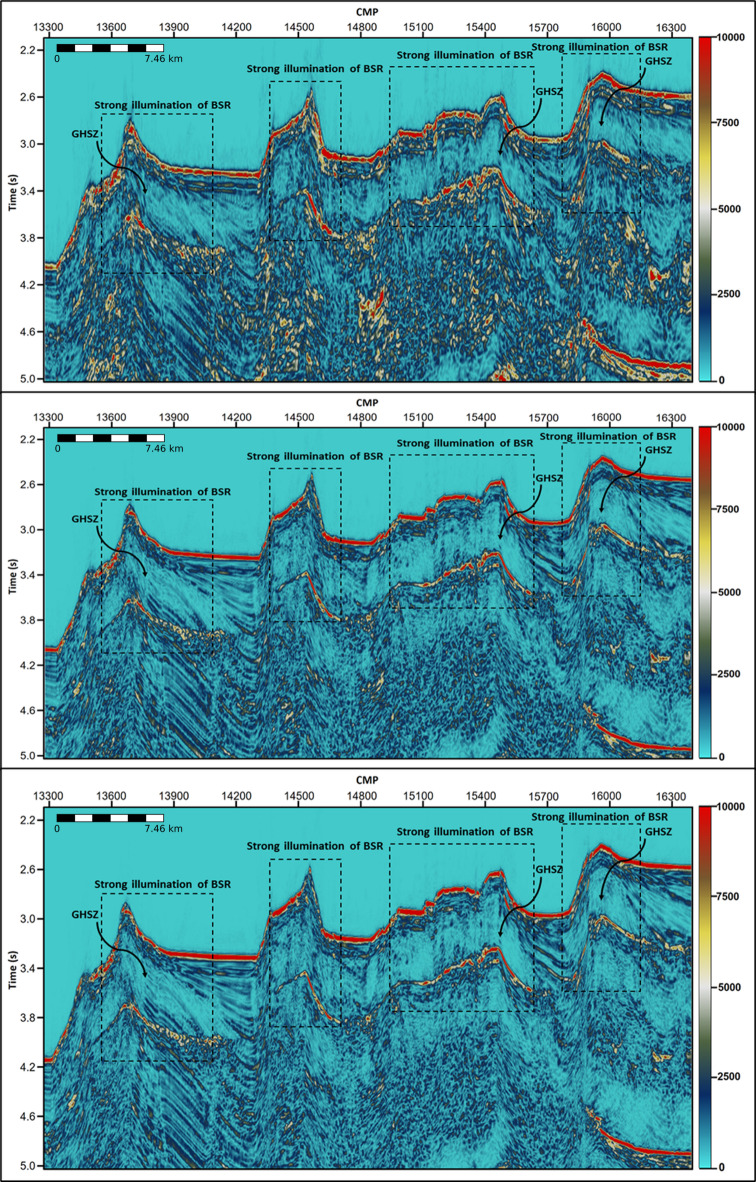
CWT-based spectral decomposition of seismic profile SO122-04a at (**a**) 20 Hz, (**b**) 40 Hz, and (**c**) 60 Hz. The 20 Hz component highlights broad BSR expression but is more affected by low-frequency background effects, whereas the 40 Hz and 60 Hz components display more localized variations in reflector expression of the BSR interval and associated anomaly patterns, illustrating time–frequency variations across the usable bandwidth.

**Fig. 12:**
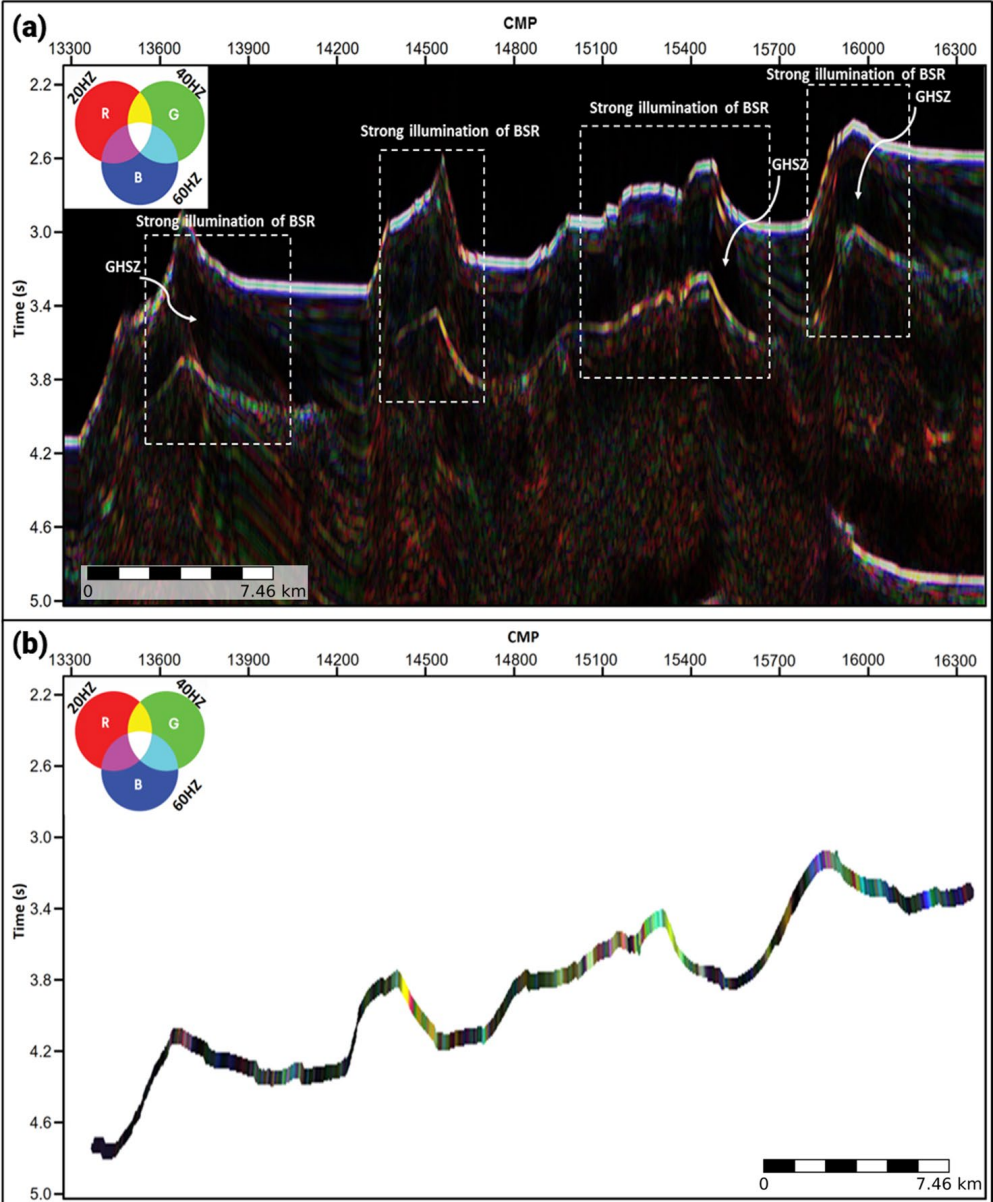
(**a**) RGB color blend of FFT-based spectral components at 20, 40, and 60 Hz to visualize the multi-frequency expression of the BSR interval on seismic profile SO122-04a. (**b**) RGB extract of the BSR interval, highlighting areas where the BSR expression is dominated by different frequency bands.

To support our interpretation and add value to the spectral decomposition analysis, we used Red-Green-Blue (RGB) color blending. The aim was to visually integrate the multi-frequency decomposition results and highlight spatial patterns in BSR-related anomalies. RGB color blends combine multiple attribute layers into a single normalized visualization, to highlight the features of interest in seismic data^58^,^59^. An RGB color blend was therefore performed using the FFT decomposition results (Fig. 11a, b) and the CWT decomposition results (Fig. 12a, b), assigning *R* = 20 Hz, G = 40 Hz, and B = 60 Hz. Prior to blending, each frequency slice was normalized independently using min–max scaling to [0, 1] to ensure comparable dynamic range across channels. Because RGB appearance depends on normalization and color mapping, we interpret RGB blends only in conjunction with the underlying single-frequency FFT/CWT results and amplitude-based attributes.

### Integration of frequency attributes

Our analysis was further reinforced by integrating all six frequency decomposition attributes (FFT- and CWT-derived components at 20, 40, and 60 Hz) using average- and median-response attributes. The average response summarizes the typical multi-frequency attribute expression across the profile, providing a compact representation of areas where frequency responses are consistently elevated or reduced. The median is the middle value of a dataset and is less affected by outliers or extreme values, making it a robust measure of central tendency when attribute responses are locally variable or skewed.

**Fig. 13:**
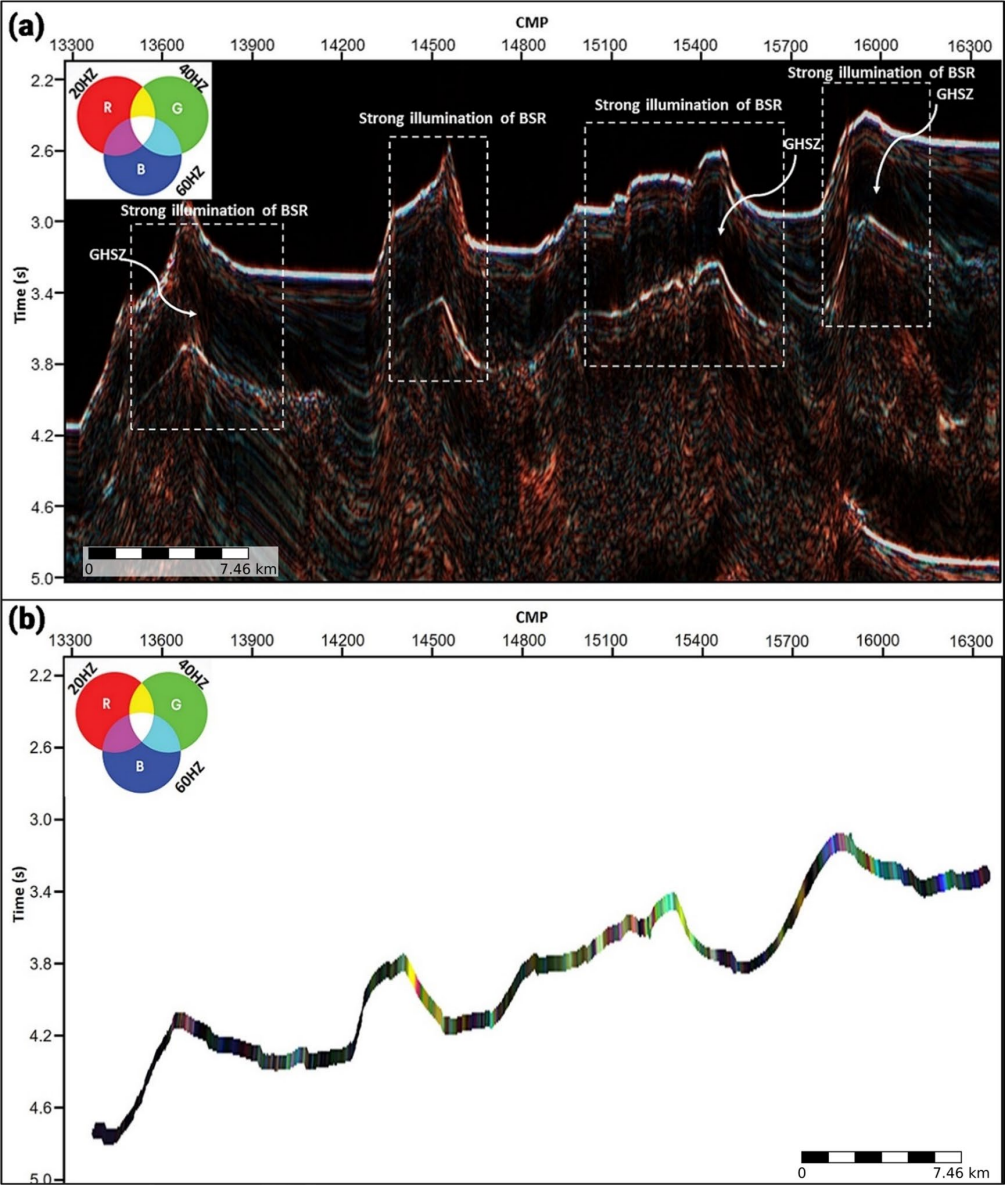
(**a**) RGB color blend of CWT-based spectral components at 20, 40, and 60 Hz applied to seismic profile SO122-04a. (**b**) RGB extract of the BSR interval, where the combined frequency blend helps visualize the continuity of BSR-related anomaly patterns.

**Fig. 14:**
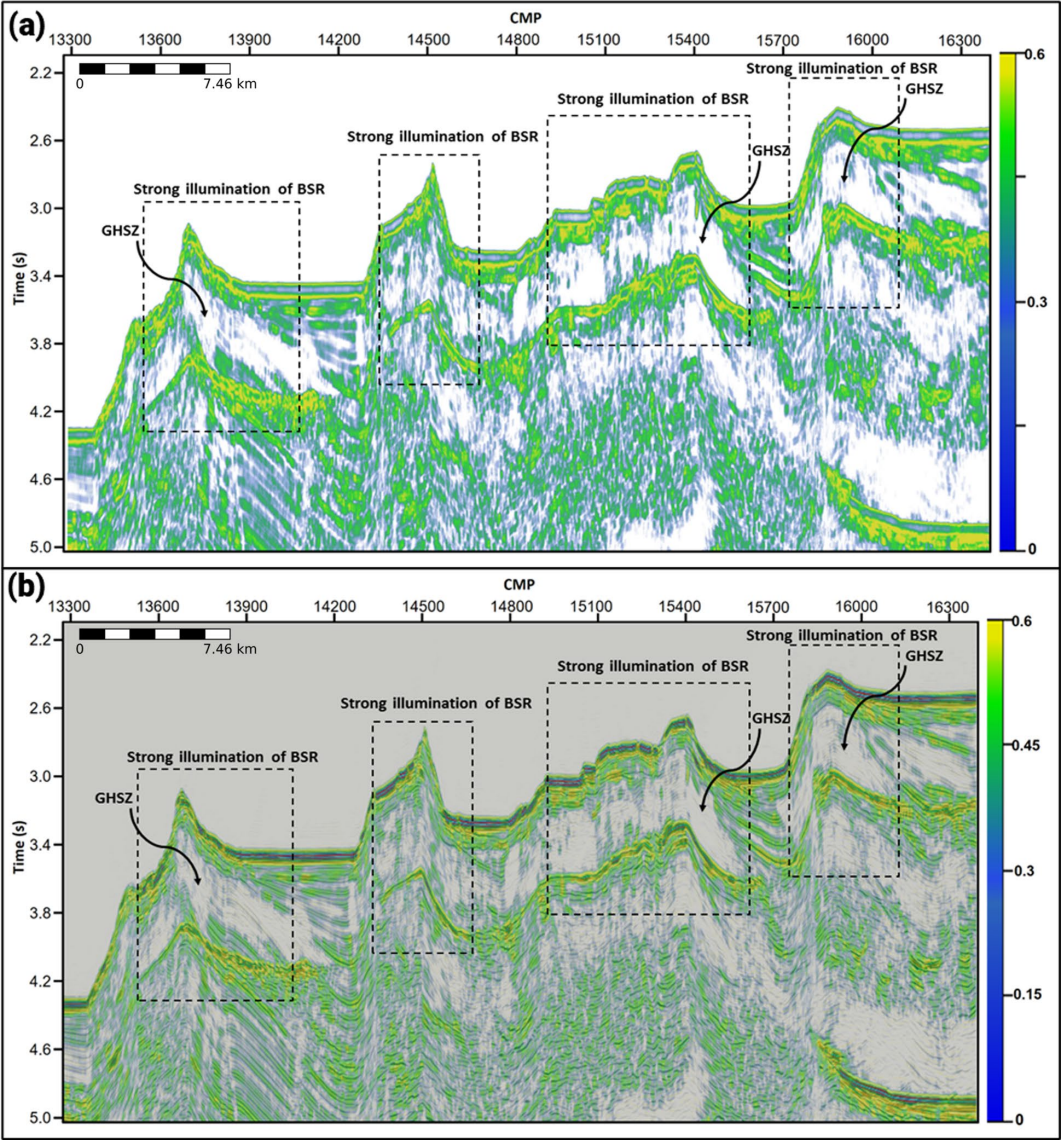
(**a**) Average-response attribute derived from FFT- and CWT-based frequency components at 20, 40, and 60 Hz for seismic profile SO122-04a. (**b**) Average-response attribute overlaid on the seismic profile, highlighting the spatial pattern of BSR-related anomaly expression along the interpreted interval.

The resulting average-response attribute is shown in Fig. 13a, with a seismic overlay depicted in Fig. 13b to relate the attribute response with the original reflection character. The median-response attribute is shown in Fig. 14a, with the corresponding seismic overlay shown in Fig. 14b.

## Results

The analyzed portion of profile SO122-04a (CMP ~ 13300–16300; ~2.2–5.0 s TWT) contains a laterally extensive BSR that broadly mimics the seafloor and is most consistently expressed across the central and eastern parts of the section, with locally weaker continuity in structurally disturbed intervals. To evaluate the seismic expression of this interval, attribute responses were analyzed over identical CMP and TWT windows for all displays to allow direct comparison. The following subsections first quantify the effect of structure-oriented pre-conditioning on incoherent energy and spectral content (Table 1; Fig. 6), and will then compare the expression of the BSR interval across amplitude- and frequency-based attributes.

Within this interval, the BSR is primarily observed between ~ 3.3–3.9 s TWT and is most continuous within the CMP ranges 13,500–14,100, 14,400–14,900, 15,000–15,600, and 15,800–16,200, while weaker expression occurs in the intervening segments. Structure-oriented conditioning using DSMF reduced incoherent energy across the section, with the median residual RMS decreasing by 29.16% in the global window and by 36.40% in the BSR-focused window. Frequency decomposition further indicates that lower frequencies (20 Hz) emphasize broader lateral continuity of the BSR expression, whereas higher frequencies (40–60 Hz) display comparatively stronger local amplitude expression and finer vertical detail within the BSR interval.

### Step 1: Application of pre-condition filters

Identifying BSRs and associated intervals commonly interpreted as part of the FGZ can be challenging in seismic interpretation of gas hydrate systems. The BSR is a negative-polarity reflection that broadly mimics the seafloor and marks the base of gas hydrate stability^7^. In many hydrate provinces, reflection packages beneath the BSR are commonly interpreted as representing a free-gas zone, often associated with reduced hydrate stability and/or focused gas charge^31^. BSRs and associated reflections can be influenced by noise and diffractions, which affect how they are resolved in seismic data.

We therefore first applied structure-oriented pre-conditioning to suppress incoherent noise and stabilize reflector continuity before computing amplitude- and frequency-based attributes. Figure 5 shows the effect of applying DSMF to SO122-04a as a data-conditioning step prior to attribute computation, which improves reflector continuity within the BSR interval and in the underlying reflection package commonly interpreted as part of the FGZ. This conditioning increases interpretability and supports more stable attribute extraction (amplitude, frequency, phase) for qualitative delineation of BSR-related anomalies and associated seismic responses.

### Step 2: Appraisal of DSMF when applied to seismic data

As illustrated by the side-by-side comparison in Figs. 5a–c (original, filtered, and difference), DSMF reduces incoherent, short-wavelength energy and improves the lateral continuity of key reflectors without introducing observable geometrical artifacts. The difference panel depicts the energy attenuated by the filter, with the strongest differences occurring in noisy intervals rather than along the main continuous reflectors (Fig. 5c). To check that conditioning did not introduce spectral boosting, we compared amplitude spectra over the same 2.2–5.0 s window before and after filtering (Fig. 6). The dominant energy band remains similar, while the conditioned data show a reduced high-frequency tail above 60 Hz, consistent with suppression of incoherent noise rather than bandwidth enhancement. This qualitative impression is consistent with the quantitative QC (Table 1). In the global window, the median residual RMS decreased from 1966.44 to 1393.08 (median reduction 29.16%). In the BSR-focused window (3.3–3.9 s below the seabed), the median residual RMS decreased from 1527.09 to 971.17 (median reduction 36.40%), showing a larger reduction in residual (incoherent) energy over the BSR interval than in the broader sub-bottom window.

Figure 7 presents the conditioned seismic profile with the interpreted BSR horizon, where the BSR can be traced across much of the line and is overlain by the interval interpreted as the gas hydrate stability zone (GHSZ). The BSR expression is laterally variable and becomes clearest within the marked CMP intervals (approximately CMP 13500–14100, 14400–14900, 15000–15600, and 15800–16200) in the 3.3–3.9 s TWT range, where the reflector is most continuous and the underlying reflection package shows a more pronounced amplitude character. These highlighted segments are therefore used as reference intervals for subsequent attribute comparisons along this profile.

### Step 3: Seismic-attribute analyses

After dip-steered median-filter conditioning (Figs. 5, 6 and 7), we computed a set of amplitude- and frequency-based attributes to examine how consistently the BSR interval and associated anomalies are expressed across different attribute families. The results below highlight reflector continuity, anomaly persistence, and frequency-dependent behavior along the BSR interval.

#### Application of amplitude-based attributes

Figure 8 shows the Pseudo-Relief attribute applied to SO122-04a. Relative to the conventional amplitude display, the Pseudo-Relief emphasizes the geometric expression of reflectors and improves the visual distinction of the BSR relative to surrounding reflections. The BSR is most clearly expressed as a laterally continuous reflector within the interpreted BSR interval, with stronger expression in the CMP ranges 13,500–14,100, 14,400–14,900, 15,000–15,600, and 15,800–16,200 within the 3.3–3.9 s time window. In contrast, weaker and more discontinuous expression is observed in the intervening CMP segments, where the BSR appears less sharply delineated and local noise or diffraction effects are more apparent.

Figure 9 shows the Square-Root-of-Energy attribute applied to the same profile. This attribute highlights spatial variations in reflection energy and improves the continuity of reflectors associated with the BSR. The BSR is emphasized by higher energy response relative to adjacent zones, and the lateral continuity of key reflectors is improved compared to the conventional display. Across the profile, the strongest responses coincide with the CMP segments where Pseudo-Relief also shows the most coherent BSR expression, with similar spatial patterns observed in both amplitude-based attributes.

#### Application of frequency-based attributes

Frequency decomposition results are depicted in Figs. 10 and 11, which show FFT- and CWT-based decompositions evaluated at 20, 40, and 60 Hz respectively. The frequencies of 20, 40, and 60 Hz were selected to represent low-, mid-, and higher-frequency components within the usable bandwidth of the seismic data, as indicated by the amplitude spectrum analysis shown in Fig. 6, allowing comparison of attribute behavior across different parts of the spectrum. The decompositions reveal frequency-dependent variations in the expression of the BSR interval and the underlying reflection package beneath it.

In the FFT results (Fig. 10), the 20 Hz component emphasizes broad, laterally continuous reflectors, supporting assessment of the lateral continuity of the BSR expression, but with reduced vertical detail (Fig. 10a). The 40 Hz component shows more distinct reflector definition relative to the lower-frequency component and highlights the BSR interval against surrounding reflectivity (Fig. 10b). The 60 Hz component displays relatively finer vertical detail among the three frequency sections, showing separation of closely spaced reflections within and near the BSR interval (Fig. 10c). The higher-frequency responses, however, are more spatially variable and locally sensitive to weaker signal or residual noise.

In the CWT results (Fig. 11), the 20 Hz component highlights the BSR interval but shows more pronounced low-frequency background effects, which reduce interpretability in noisier portions of the profile (Fig. 11a). The 40 Hz and 60 Hz components show a more distinct expression for the BSR interval, with a more localized representation of reflector geometry and time-frequency character relative to the lower-frequency components (Fig. 11b,c). Overall, comparison of the FFT and CWT figures (Figs. 10 and 11) indicates that higher-frequency components (40–60 Hz) tend to locally display greater detail within the BSR interval, whereas the lower-frequency component (20 Hz) highlights broader lateral continuity, but provides less vertical detail.

#### RGB color blending of decomposed frequency components

In RGB composites (Figs. 12 and 13), the three frequency components are combined into a single visualization to examine the persistence of BSR-related patterns across the frequency bands. In the FFT-based RGB blend (Fig. 12), the BSR interval is expressed as a multi-frequency anomaly pattern, with local color variations indicating shifts in the dominant frequency contribution along the profile. In the CWT-based RGB blend (Fig. 13), the BSR interval appear visually more continuous with localized color variations reflecting contributions from different frequency bands. In both cases, the RGB composites summarize multi-band behavior in a single display and complement the single-frequency decomposition results (Figs. 10 and 11) rather than serving as standalone evidence.

#### Average- and median-response attributes

**Fig. 15:**
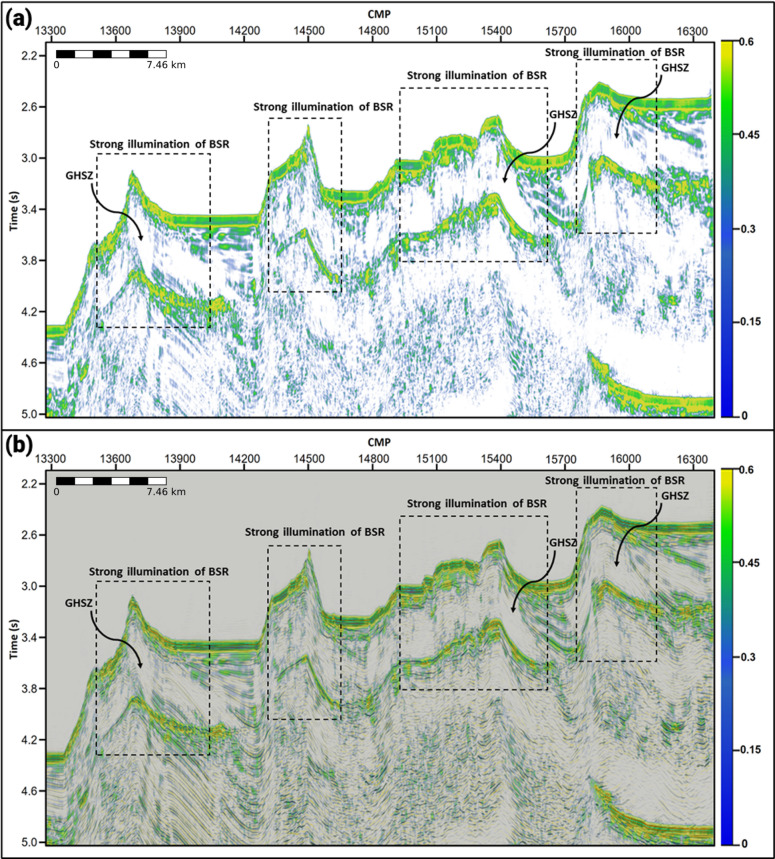
(**a**) Median-response attribute derived from FFT- and CWT-based frequency components at 20, 40, and 60 Hz for seismic profile SO122-04a. (**b**) Median-response attribute overlaid on the original seismic profile, highlighting persistent BSR-related anomaly patterns along the interpreted interval.

We further evaluated multi-frequency behavior by computing average- and median-response attributes from the FFT and CWT frequency components (Figs. 14 and 15). The average-response attribute displays broader spatial patterns across the BSR interval and highlights where frequency responses are consistently prominent across bands (Fig. 14). The median-response attributes highlight persistent anomaly patterns while limiting the influence of extreme values that can bias the mean (Fig. 15). Together, these summaries reproduce the main BSR-related anomaly patterns observed in the single-frequency FFT/CWT results and amplitude attributes, while providing a profile-scale summary of these patterns.

## Discussion

This study is based on a single 2D seismic profile and lacks independent calibration data along the interpreted BSR interval (such as wells/cores, pore-water geochemistry, or rock-physics/velocity inversion constraints). Interpretations of gas hydrate occurrence and underlying free-gas therefore remain qualitative and are presented as seismic indicators consistent with gas hydrate/free-gas systems rather than quantitative estimates of hydrate saturation or concentration. Extending these interpretations to geohazard assessment would require additional datasets and broader spatial coverage. Spectral balancing methods (e.g., whitening/bluing) may further increase apparent resolution; however, they were not applied here to avoid potential bias in amplitude- and frequency-based attribute comparisons given the limited high-frequency bandwidth and lack of independent calibration.

### Applicability of amplitude, frequency, and color-blending attributes in the identification of BSRs in seismic data

Figure 8 shows that amplitude-based attributes support the interpretation of the BSR and associated seismic anomaly patterns in the BSR interval offshore Makran. In Fig. 8, Pseudo-Relief clearly highlights the BSR within CMP ranges 13,500–14,100, 14,400–14,900, 15,000–15,600 and 15,800–16,200, within a 3.3–3.9 s time window, supporting a more confident qualitative interpretation. Furthermore, a prominent BSR expression is locally associated with blank zones (of relatively low relief) immediately above and below; however, such dimming/blanking is non-unique and can also arise from tuning/interference, scattering, residual noise, or processing/migration effects, and is therefore treated here as a supporting observation rather than a diagnostic indicator of hydrate concentration or free-gas saturation (Fig. 8). Zones with a more prominent BSR expression, together with lower illumination immediately above and below (dim zones), may indicate lateral variability in the seismic expression of the BSR but cannot be uniquely attributed to attenuation effects with the available 2D post-stack data. Where a strong BSR is associated with underlying reflections interpreted as possible free-gas indicators beneath the BSR, this pattern is consistent with seismic responses reported for hydrate/free-gas systems in previous studies.

This interpretation is supported by the Square-Root-of-Energy attribute, which shows a similar trend to Pseudo-Relief by showing higher energy values for the BSR; the implication being that higher Square-Root-of-Energy values correspond to greater reflection amplitudes of the strata with the BSR (Fig. 9). This attribute highlights the lateral continuity of seismic events and is thus valuable in seismic-object detection (Fig. 9).

Frequency-based attributes return varied results depending on the frequency used. At 20 Hz, the BSR shows good continuity (Fig. 10), meaning that the BSR reflection is well-defined and can be traced without significant interruptions, although the event character remains relatively broad in the vertical direction. At 40 Hz, the BSR reflection appears cleaner and more coherent, consistent with suppression of surrounding incoherent noise within this band (Fig. 10b). At 60 Hz, the BSR illumination is not markedly strong using the FFT-based attribute, but the attribute still helps to delineate reflector terminations and thin-bed-scale events in the intervals interpreted at CMP ranges 13500–14100, 14400–14900, 15000–15600 and 15800–16200 (Fig. 12). Overall, the results indicate that an FFT at a low-frequency band (20 Hz) is helpful to investigate the lateral continuity of strongly expressed BSR segments, while the high-frequency bands (40 and 60 Hz) appear to suppress incoherent noise more effectively and separating closely spaced reflectors. This trade-off suggests that attribute interpretation should rely on consistent patterns across multiple bands rather than any single frequency slice.

For spectral decompositions using a CWT, our results at 20 Hz show a strong expression of the BSR but this is accompanied by a lower apparent S/N ratio (Fig. 11a), and the BSR character is partly influenced by adjacent and overlapping noise. In contrast, the 40 Hz and 60 Hz display clearer local expression of the BSR interval in the section (Fig. 11b,c). Only minor differences are observed between the 40 Hz and 60 Hz displays, suggesting that both frequencies may provide a comparable visualization of BSR expression and associated reflections interpreted as part of the FGZ. Overall, these results illustrate how spectral decomposition, when applied across different frequency bands, can help reveal frequency-dependent variations in BSR-related anomalies on continental margins.

Our RGB (Red-Green-Blue) color blending was helpful in mapping BSR continuity. The resulting composite images in Figs. 12 and 13 highlight trends and changes in the spatial expression of BSR-related anomalies and hydrate indicators caused by the blending of these three-color channels at 20 Hz, 40 Hz, and 60 Hz. We observe that the color blending provides clearer visualization of the BSR and underlying reflections interpreted as free-gas, supporting interpretation when considered alongside the individual frequency panels (Figs. 12 and 13). Because RGB blending is sensitive to color mapping and relative scaling of the input volumes, we interpret these composites as visualization aids that summarize multi-frequency patterns, not as standalone evidence of hydrate presence. In Fig. 12a, the FFT-based RGB blending illustrates the BSR expression by utilizing 20, 40, and 60 Hz frequency bands. Each frequency contributes distinctively and aids the interpretability of subtle features in the data. Figure 12b further illustrates the BSR intervals, showing clearer delineation of areas dominated by individual frequencies. Figure 13 depicts a CWT-based RGB blending. Compared with FFT, the CWT method provides a more continuous representation of the signal across the same frequency bands (Fig. 13a), and it highlights variability within the BSR while providing a more continuous representation of the seismic response. Figure 13b highlights regions where the BSR is prominent by combining the contributions of each frequency band.

In addition to these pointwise attributes, we used average and median summaries to stabilize attribute responses across the BSR interval and reduce the influence of extreme values. As attribute responses vary across the BSR intervals at multiple locations, integrating frequency attributes summarizes the average multi-frequency attribute response. This summary provides interpreters with a representative measure of attribute expression and helps describe broader spatial patterns of BSR-related anomalies across the profile (Fig. 14a,b). In fact, the median response attribute gives a representative value that differentiates the higher and lower levels of attribute expression, which is especially beneficial where the dataset contains extreme values that may disproportionately influence the average (Fig. 15a,b). These summaries support clearer visualization and interpretation of gas hydrate indicator patterns, particularly in areas with high signal variability (Fig. 15a,b).

Attribute responses across the BSR interval should be interpreted carefully because they can be influenced by tuning, local changes in wavelet character, residual noise, and processing artifacts, e.g., migration or stacking effects, not only by subsurface fluid changes. Furthermore, frequency slices may also reflect bandwidth limitations and interference between closely spaced reflectors. This could cause an apparent sharpening at higher frequencies partly from reduced low-frequency smearing rather than true geological variation. Similarly, amplitude blanking can result from attenuation or scattering and is not unique to hydrates alone. These considerations reinforce that presented attributes provide qualitative indicators and comparative assessments rather than definitive validation.

### Use of average- and median-response attributes in the analysis of BSRs and other hydrate-related anomalies

Seismic attributes have been successfully applied for the analysis of the BSR and affiliated shallow gas accumulations^60^. Information regarding discontinuities and other resolvable features can be extracted from seismic data using seismic attributes from the raw seismic amplitudes^61^. For instance, the Energy attributes such as average energy provide information regarding the lowest and highest energy reflectors highlighting the lithological variations otherwise difficult to interpret on the seismic data^62,63,64^. This work shows that the Pseudo-Relief attribute can highlight BSR expression by emphasizing curvature and geometry (Fig. 8). In parallel, Square-Root-of-Energy highlights spatial variations in reflection energy that are consistent with gas hydrate indicator zones along the BSR interval (Fig. 9).

In many hydrate provinces, BSR-related intervals may show reduced amplitudes and higher apparent frequencies above the BSR, whereas bright amplitudes and lower apparent frequencies beneath the BSR are commonly interpreted as possible indicators of free-gas (e.g., ^24^). Knowing this, seismic data can be decomposed into a band of frequencies and specific frequency bands can be extracted. Spectral decomposition attributes convert the signal to the frequency domain, providing a frequency-based analysis of the subsurface. For instance, ^65^ applied CWT spectral decomposition to analyze seismic frequency content and inferred gas hydrates beneath the BSR based on high energy and low frequency values. In parallel, ^26^ applied short-time Fourier transform, CWT, and matching pursuit decomposition in the Ulleung Basin, Korea, and concluded that these decomposition attributes are beneficial in highlighting zones with BSRs and associated gas hydrates. Finally, ^66^ observed the impact of gas hydrate presence on local attenuation by statistically analyzing waveforms in BSR regions in the Pegasus Basin, offshore New Zealand. They deemed the FFT-based amplitude and phase components critical in interpreting possible indicators of free-gas and gas hydrates, respectively, below and above the BSR. However, in the absence of calibration data along SO122-04a, we use these relationships only as interpretive guidance.

In our work, FFT and CWT-based decomposition returned frequency-dependent variations in the expression of the BSR interval, with the 40 Hz and 60 Hz bands providing more distinct expression of BSR-related anomalies and underlying reflections interpreted as possible free-gas indicators (Figs. 10 and 11). A practical advantage of this approach is that it allows interpreters to compare how reflector expressions change across low and higher frequency bands within the usable bandwidth (Figs. 10 and 11). However, when applying attributes to increase the prominence of geological features on seismic profile SO122-04a, an important learning from our work is that plausible interpretation is strongly influenced by color perception^67^. This motivated the color blending of multi-dimensional seismic attributes completed in this work (see ^67,68^). Our RGB co-blending produced informative multi-attribute visuals, thereby aiding interpretation and allowing the integration of information from multiple sources using a single visual representation. In addition, ^69^ combined the decomposed frequency bands of seismic data with a RGB blending to reduce the coherent and random noise, thereby revealing a wide range of geological features on the seabed and in the subsurface.

We applied the RGB color blending technique to integrate the spectral decomposition results. The RGB co-blending combines three representative frequency slices (*R* = 20 Hz, G = 40 Hz, B = 60 Hz) derived from FFT and CWT. The resulting composite images highlight changes in the spatial expression of BSR-related anomalies and gas hydrate indicators, aiding visual interpretation of frequency-dependent patterns along the BSR interval (Figs. 12 and 13). Color blending supports the interpretation of the BSR geometry and underlying reflections interpreted as possible free-gas indicators by combining complementary information from multiple frequency bands in a single display (Figs. 12 and 13).

The FFT-based RGB blend shows the frequency expression across the three bands (*R* = 20 Hz, G = 40 Hz, B = 60 Hz) and highlights BSR-related features by emphasizing frequency-dependent contrasts. Its global nature, however, limits its ability to capture refined local variations (Fig. 12). Comparatively, the CWT-based RGB blend in Fig. 13 captures more localized variations because it represents time-frequency behavior, providing a smoother and more continuous depiction of BSR expression and fine-scale signal variability. While FFT is computationally efficient and well suited for identifying broad trends, CWT provides more localized time-frequency detail in this dataset.

By integrating all six frequency attributes, the attribute analysis was reinforced further by the addition of average-response attributes and median-response attributes (Figs. 14 and 15). The average response attribute offers insight into the typical multi-frequency attribute response along the profile and helps describe broader spatial patterns in BSR-related anomalies and gas hydrate indicators (Fig. 14a and b). The median response attribute, being less influenced by outliers, provides a robust measure of central tendency when attribute responses show high variance or skewness. In this context, the median values help distinguish higher and lower levels of attribute expression, which can be particularly useful when extreme values could disproportionately influence the mean response. Overall, these attributes support a more stable qualitative interpretation of the BSR interval by emphasizing persistent anomaly patterns (Figs. 14 and 15).

Overall, the average-response attributes are well suited to interpreting broad-scale patterns in attribute expression along the BSR interval, whereas the median-response attributes highlight more persistent and stable patterns in the presence of local variability (Fig. 15). The results can be compared across CMP ranges (13500–14100, 14400–14900, 15000–15600, and 15800–16200) within the 3.3–3.9 s time window (Figs. 8, 9, 10, 11, 12, 13, 14 and 15). Across the suite of attributes, the BSR is consistently expressed as a distinctive feature, although its apparent illumination strength varies laterally. More prominent expressions are observed at CMP ranges 13,500–14,100, 14,400–14,900, 15,000–15,600, and 15,800–16,200, whereas weaker expressions occur at CMP ranges 13,300–13,499, 14,101–14,399, 14,901–14,999, 15,601–16,199, and 16,201–16,500. These variations may reflect lateral changes in seismic attenuation, tuning, or local noise conditions across the BSR interval. Notably, low-frequency “shadow” effects coincide with reduced amplitudes in places, and their recurrent appearance across multiple amplitude- and frequency-based attributes supports interpretation of laterally variable BSR-related anomaly patterns. Overall, the attribute suite helps to characterize spatial variability in BSR expression and associated seismic indicators in the absence of independent calibration data. Taken together, the workflow is most useful for screening and mapping where BSR expression is laterally persistent and where anomaly patterns recur across independent attribute families, which reduces reliance on any single, potentially misleading attribute.

## Conclusions

Several observations arise from the attribute analysis performed in this work. The application of amplitude- and frequency-based attributes to the resolution-enhanced seismic profile SO122-04a, which contains a laterally continuous BSR previously interpreted offshore Makran, supports the following conclusions:

1. The amplitude-based Pseudo-Relief and Square-Root-of-Energy attributes highlight the BSR interval and associated seismic anomaly patterns. The Pseudo-Relief attribute shows stronger illumination at CMP ranges 13,500–14,100, 14,400–14,900, 15,000–15,600, and 15,800–16,200 within a 3.3–3.9 s time window, whereas weaker illumination occurs at CMP ranges 13,300–13,499, 14,101–14,399, 14,901–14,999, 15,601–16,199, and 16,201–16,500. These lateral variations may reflect changes in seismic attenuation, tuning, or local noise conditions across the BSR interval. The Square-Root-of-Energy attribute emphasizes high-energy reflections associated with the BSR, highlighting the lateral continuity of key reflectors.
2. Frequency decompositions using FFT and CWT at 20, 40, and 60 Hz demonstrate complementary strengths of lower and higher frequency bands. The 20 Hz band is useful for assessing lateral continuity of the BSR expression but is more affected by low apparent S/N ratio and reduced vertical detail. The 40 and 60 Hz bands display greater local detail in the BSR and in underlying reflections interpreted as possible free-gas indicators, with CWT generally offering more localized time-frequency detail compared to FFT.
3. Average- and median-response attributes derived from FFT and CWT spectral decompositions further summarize multi-frequency attribute expression across the BSR interval. The average response captures broader trends in BSR-related anomaly patterns along the profile, whereas the median response highlights persistent patterns while reducing sensitivity to extreme values. In the absence of independent calibration data, these attributes support qualitative mapping of BSR-related anomalies rather than quantitative estimates of hydrate saturation, concentration, or distribution.

## Data Availability

The data used in this work can be obtained from Directorate General of Petroleum Concessions (DGPC) in Pakistan for research purposes by submitting a formal request to the data management section of such directorate. The DGPC is authorized to share seismic and well data with the third party. Normally the data permission is granted upon formal request - as the data used in this paper is public domain data.
